# Association between maternal exposure to indoor air pollution and offspring congenital heart disease: a case–control study in East China

**DOI:** 10.1186/s12889-022-13174-0

**Published:** 2022-04-15

**Authors:** Jing Sun, Jian Wang, Jing Yang, Xin Shi, Shujing Li, Jinping Cheng, Sun Chen, Kun Sun, Yurong Wu

**Affiliations:** 1 Department of Pediatric Cardiology, School of Medicine, Xinhua Hospital, Shanghai Jiao Tong University, No. 1665 Kongjiang Road, Shanghai, 200092 China; 2 Department of Fetal Echocardiography, Jiaxing Maternity and Child Health Care Hospital, No. 2468 Zhonghuan Dong Road, Jiaxing, 314051 China; 3 School of Environmental Science and Engineering, Shanghai Jiao Tong University, No. 800 Dongchuan Road, Shanghai, 200240 China

**Keywords:** Household indoor air pollution, Congenital heart disease, Volatile organic compounds, Particulate matter, Maternal personal exposure

## Abstract

**Background:**

Previous research suggested an association between maternal exposure to ambient air pollutants and the risk of congenital heart disease (CHD). However, the effect of individual prenatal exposure to indoor air pollutants on CHD occurrence was not reported.

**Methods:**

We performed a hospital-based case–control study to investigate the association between personal air pollution exposure during pregnancy and the risk of CHD in offspring. A total of 44 cases and 75 controls were included from two hospitals in East China. We investigated maternal and residential environmental characteristics using a questionnaire and obtained personal indoor air samples to assess particulate matter (PM) and volatile organic compounds (VOCs) from 22–30 gestational weeks. Formaldehyde, benzene, toluene, xylene, total volatile organic compounds (TVOCs), PM_2.5_, and PM_10_ were assessed. Logistic regression was performed to assess associations and interactions between individual indoor air pollutants and CHD after adjusting for confounders. The potential residential environmental factors affecting the risks of indoor air pollutants on CHD were also assessed.

**Results:**

Median TVOC (0.400 vs. 0.005 mg/m^3^, *P* < 0.001) exposure levels in cases were significantly higher than controls. A logistic regression model adjusted for confounders revealed that exposure to high levels of indoor TVOCs (AOR 7.09, 95% CI 2.10–23.88) during pregnancy was associated with risks for CHD and the occurrence of some major CHD subtype in offspring. These risk effects were enhanced in pregnant women living in a newly renovated house but were mitigated by household use of smoke ventilators when cooking. We observed a positive interaction of maternal exposure to TVOCs and PM_2.5_ and the risk for CHD.

**Conclusions:**

Maternal exposure to indoor VOCs and PMs may increase the risk of giving birth to foetuses with CHD.

**Supplementary Information:**

The online version contains supplementary material available at 10.1186/s12889-022-13174-0.

## Background

Congenital heart disease (CHD) is the most common congenital anomaly and a leading cause of infant death [1, 2]. There are a number of common factors that affect CHD, including the protective effect of periconceptional folic acid supplementation [3] and risk factors, such as advanced maternal age [4], low socioeconomic status [5], maternal diabetes mellitus [6], and maternal smoking and alcohol consumption [7]. An increasing amount of epidemiological evidence suggests associations between maternal exposure to ambient air pollution and cardiovascular malformations in offspring [8–11]. With increases in urbanization and improvements in living standards in developed eastern China, people's living environments and lifestyles have undergone significant changes, which may lead to the emergence of novel risk factors for CHD in this population.

The public is paying great attention to indoor air quality due to elevated indoor chemical concentrations and because most of people’s time is spent in indoor environments. Exposure to indoor air pollution is linked to cardiovascular [12] and pulmonary [13] effects. Individuals are exposed to various indoor pollutants from outdoor and indoor-specific sources, such as oil and gas combustion, transportation emissions, decoration materials and pollutants associated with various activities, such as cooking, cleaning and biological emissions, which emit particulate matter (PM) and volatile organic compounds (VOCs) [14–17]. VOCs, such as formaldehyde, benzene, toluene, xylene (BTX), and total VOCs (TVOCs), are released primarily from paints and adhesives used in building and decorative materials and indoor and outdoor oil and gas combustion, which result in adverse health outcomes [18]. PM less than 10 and 2.5 microns in diameter (PM_10_ and PM_2.5_) penetrate deep into the lungs and enter the bloodstream. Therefore, epidemiological evidence suggests that exposure to PM_10_ during the first trimester of gestation may increase the risk of CHD [19].

Pregnant women and their foetuses are at higher risk than the normal population because women tend to spend more time indoors during pregnancy and are more susceptible to environmental toxicants. Although the rapidly growing epidemiological literature reports associations between atmospheric pollutants and adverse pregnancy events [8, 10, 20], there are limited data on the link between prenatal exposure to indoor air pollutants and CHD. Cardiac development is vulnerable to various physical and chemical factors that may lead to heart developmental abnormalities within the first 8 weeks of gestation. The risk for CHD in offspring is significantly associated with maternal periconceptional housing renovation exposure, which is a newly recognized source of indoor environmental pollution [21].

## Methods

### Study population and subjects

We performed a hospital-based case–control study from May 2017 to May 2021 at two perinatal medical centres in East China (Shanghai Xinhua Hospital and Jiaxing Maternity and Child Health Care Hospital). These two centres are qualified as regional prenatal diagnosis centres with high-level ultrasound technology for the detection of foetal defects [22]. The study population was recruited from the population of pregnant women undergoing foetal echocardiography in the Department of Foetal Echocardiography in these two centres. Mothers receiving treatment in the hospital's obstetrics department are referred to the Foetal Echocardiography Unit when CHD is suspected after an obstetric ultrasound examination. Only participants who gave voluntary written informed consent to participate in the study were included in the survey. The inclusion criteria for the study population were (1) singleton pregnancy, (2) gestational age between 22 and 30 weeks at the time of the prenatal diagnosis, and (3) undergoing a thorough foetal echocardiography. Foetuses with chromosomal or other genetic syndromes diagnosed after referral to the clinical genetics service were excluded from our study. CHD cases that were associated with other congenital extracardiac defects were also excluded. The case group included (1) foetuses diagnosed with a defined isolated CHD and (2) all foetal heart diseases and malformations confirmed after birth or abortion. Infants who were stillborn with CHD (including miscarriages and elective pregnancy terminations resulting from CHD) were eligible for inclusion if the diagnosis had been made prenatally. The controls were selected from the same hospital during the same study period as the cases with a general case–control ratio of 1:2 and had no more than a two-week difference in gestational age compared with the case group. Infants in the control group were defined as foetuses without CHD or other congenital malformations. A multidisciplinary team composed of experienced obstetricians, ultrasonic physicians, and paediatricians made every diagnosis.

All live births in the CHD case and control groups underwent complete neonatal echocardiography by paediatric cardiologists after delivery. An expert group composed of 4 national specialists from the fields of ultrasound, paediatrics, obstetrics and pathology reviewed stillbirth and abortion cases to ensure the accuracy of the final diagnosis.

**Fig. 1:**
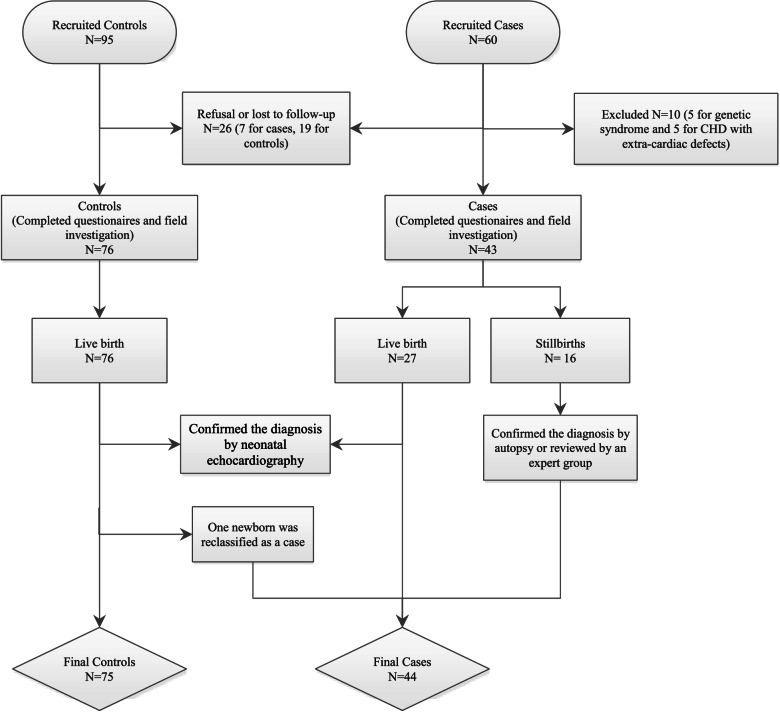
The flow chart of study population recruitment

The recruited sample included 60 cases and 95 controls, including 7 cases and 19 controls who refused to participate or were lost to follow-up (Fig. 1). Five cases with a genetic syndrome and 5 cases with extracardiac defects were excluded, and 43 cases remained. Seventy-six infants were live born with diagnoses confirmed by echocardiogram, and one infant in the control group was diagnosed with pulmonary stenosis and reclassified into the case group. Therefore, 44 cases and 75 controls were ultimately available for analysis in our research.

All CHD cases were classified into subtypes based on anatomical malformation as previously proposed [23]. Because multiple anatomical abnormalities may coexist in a single case, these groupings were not mutually exclusive, and overlap may exist. The following subtypes were described: (i) septal defects (*n* = 21), including ventricular septal defects, atrial septal defects and endocardial cushion defects; (ii) conotruncal defects (*n* = 12), including tetralogy of Fallot, common truncus, transposition of the great arteries and double outlet right ventricle; (iii) right-sided obstruction (*n* = 15), including pulmonary valve stenosis, pulmonary artery/valve atresia and right ventricular hypoplasia; (iv) left-sided obstruction (*n* = 10), including coarctation of the aorta and left ventricular hypoplasia; and (v) other cardiac structural abnormalities (*n* = 4), including single ventricle and anomalous pulmonary venous return.

### Information collection

Trained researchers investigated the prenatal characteristics of the mothers using questionnaires on sociodemographics, reproductive history and periconceptional health status, including maternal and paternal age, maternal and paternal education level, maternal residence area, parity, family history of CHD, perinatal diseases (e.g., diabetes), maternal smoking, maternal alcohol consumption, and the use of folic acid and multivitamin supplements.

Information on residential environmental factors, including time spent indoors each day during the first trimester of pregnancy, indoor combustion sources, maternal exposure to indoor renovations, ventilation time in newly renovated houses, decorative materials in dwelling places, exposure to environmental pollutants around the residence, smoke ventilator use when cooking, second-hand smoke exposure (paternal smoker or other nearby smokers) and exposure to household chemical products, was also obtained from self-reported questionnaires.

### Indoor environmental field investigation and air pollutant measurements during pregnancy

Participants underwent a field investigation within two weeks after signing the informed consent form and completing the designated questionnaires. The number of gestational weeks at the time of the field investigation was recorded. This study was performed in residential dwellings in Shanghai city and its ambient area from May 2017 to May 2021. Dwelling-related self-report questionnaires were verified using a field survey. The concentrations of formaldehyde, BTX, TVOCs, and PM in households were monitored.

Room doors and windows were kept closed for 12 h before the sampling process. We chose the living room, where people spend the most time, as the sampling area for health risk assessment in pregnant women. The samplers were placed in the middle of the sampled rooms at a height of 1.5 m above the floor and 1 m away from walls to simulate the breathing zone. Indoor temperature and relative humidity were recorded when the samples were obtained.

VOCs were collected using active samplers (Tenax-TA, SUPELCO, USA) connected to a personal pump (DDY-1.5, Xingyu, China) for 20 min at a speed of 5 L/min. VOCs were analysed using gas chromatography with flame ionization detection (GC-FID). The quantification of target VOCs (BTX) was accomplished using multipoint external standard curves. The total quantified VOCs were included for TVOC concentration analysis.

Formaldehyde was sampled at a rate of 0.5 L/min for 20 min using an air sampling pump and analysed using the 3-methyl-2-benzonthiazolinone hydrazine (MBTH) method with a UV–VIS spectrophotometer at an absorption wavelength of 630 nm (WFJ7200, Shanghai Unico Instrument Co., Ltd., Shanghai, China). More information related to the sampling and analytical methods are detailed in the Chinese National Standard GB/T 18,883–2002.

The levels of PM_2.5_ and PM_10_ were determined using a DUST-TRAK Aerosol Monitor (Model 8520, TSI Corporation, Shoreview, USA). Measurements were taken every minute for 30 min, and the averages were taken to represent the PM_2.5_ and PM_10_ at the sampling area.

The method detection limits (MDLs) were 0.01 mg/m^3^ for BTX, formaldehyde and TVOC concentrations and 1.0 µg/m^3^ for PM_2.5_ and PM_10_. Observations below the MDL were replaced with half the detection limit for statistical analyses.

### Statistical analysis

Statistical analyses were performed using SPSS software (version 19.0; SPSS, Inc. IBM, Chicago, IL, USA). Data are presented as the means (standard deviation, SD), medians (interquartile) or number (%) as appropriate. Differences between groups were assessed using t tests (for normally distributed variables), Mann–Whitney U tests (for variables without a normal distribution) and chi-squared tests (for categorical variables).

Bivariate analysis was first performed for each potential CHD risk factor, including sociodemographic, reproductive history and periconceptional health status factors, and residential environmental factors, to select potential confounders for inclusion in the subsequent multivariate logistic analysis.

Because of the skewed distribution of indoor air pollutant concentrations, these concentrations were categorised into low (1^st^ tertile), middle (2^nd^ tertile) and high levels (3^rd^ tertile) based on tertile distribution of controls. The associations between indoor air pollutant exposure levels and risks of CHD were assessed by calculating the crude odds ratio (COR) and its 95% confidence interval (CI) using univariate logistic regression for each pollutant separately. Correlations between the pollutant concentrations were assessed and checked using Spearman rank correlation coefficients. Single-pollutant adjusted odds ratios (AORs) were adjusted for potential confounding effects using multivariate logistic regression. We selected confounders on the basis of the results of the bivariate analysis. Variables with a *P* value less than 0.05 were entered into multivariable logistic regression. Due to the limited number of cases in the CHD subgroups, we recategorized TVOC, formaldehyde and PM_s_ exposure levels as “low” (1^st^ and 2^nd^ tertiles) and “high” (3^rd^ tertile). These binary variables were used to estimate AORs to show the risk for CHD subtypes.

To further clarify whether residential environmental factors affect the risk of CHD at different pollutant exposure levels, factors including indoor renovations, residential surroundings, and smoke ventilator use were stratified into their respective categories, in which the AOR for CHD in mothers exposed to low-level pollutants was compared with mothers exposed to high-level pollutants. The Benjamin-Hochberg method was adopted to control the false positives and adjust *p* values were calculated.

A potentially relevant interaction between indoor air pollutants and CHD risk was evaluated by measures of effect modification on additive and multiplicative scales. TVOCs, formaldehyde and PM_2.5_ were categorised as mentioned previously (“low” for the 1^st^ and 2^nd^ tertiles, “high” for the 3^rd^ tertile). The effects were analysed for low TVOCs with low PM_2.5_, high TVOCs with low PM_2.5_, low TVOCs with high PM_2.5_, and high TVOCs with low PM_2.5_. Expected ORs in the multiplicative model were calculated as the product of the main effects. Relative excess risk due to interaction (RERI) was used to evaluate additive interaction, which was calculated for binary variables as RERI_IRR_ = IRR_11_ − IRR_10_ – IRR_01_ + 1 [24]. We assessed the presence of interactions on the additive scale using the RERI and the attributable proportion (AP) using the algorithm of Andersson et al. [25]. When the 95% CI of RERI and AP did not contain 0, an additive interaction occurred. When the *P* value of the cross-product term in the logistic model was < 0.05, a multiplication interaction occurred [26].

PASS v.19 (NCSS, LLC, Kaysville, UT, USA) was used to calculate the sample size. We assumed a TVOC high-level-exposure rate of 20% in the control group and 60% in the case group, which referenced the reported rate of indoor VOCs in China exceeding the standard rate of 20 ~ 60% [27]. The total sample size was 108 (72 controls and 36 cases), which was required to achieve 80% power for detecting different TVOC exposures between the groups with an alpha error of 0.05.

## Results

### Basic characteristics of the study subjects

The maternal sociodemographic characteristics, reproductive history and periconceptional health status of the study population are listed in Table S1 (Supplementary Information). Paternal age and parental education levels were significantly different between the case and control groups. No family history of CHD was observed in the study subjects.

### Residential environmental characteristics of the study subjects

Table 1 compares the two groups according to residential environmental characteristics. The two groups spent a similar amount of time indoors each day during the first trimester, with a median of more than 18 h per day. Significant differences in proportions between the mothers from the case and control groups were detected for maternal exposure to environmental pollutants near the residence, indoor renovations, ventilation time, and smoke ventilator use when cooking. Nine women (20.5%) reported exposure to housing renovations with a moving-in interval of less than 3 months, and the same situation occurred in only 2.7% of the control group. A larger proportion of cases than controls lived in residences near heavily trafficked roads (40.9% vs. 33.3%). The rate of ventilator use when cooking was much higher in the control group than the case group (81.3% vs. 59.1%). There were no between-group differences found in indoor combustion sources, decorative materials of dwelling places, exposure to household chemical products or exposure to second-hand smoke.

**Table 1:** Residential environmental characteristics of the study subjects

**Characteristics**	Controls (*N* = 75)	Cases (*N* = 44)	*p* Value
	n (%) or median (interquartile)	n (%) or median (interquartile)	
**Gestational weeks at the time of questionnaires (weeks) **^**a**^	24 (10)	24 (4)	0.998
**Gestational weeks at the time of field investigation (weeks) **^**a**^	26 (8)	25 (3)	0.398
**Time spent indoors each day during the first trimester (hours) **^**a**^	20 (5)	18 (5)	0.850
**House renovation and ventilation**			
No renovation	54 (72.0)	30 (68.27)	0.002
Living in a newly redecorated house with move-in interval < 3 months	2 (2.7)	9 (20.5)	
Living in a newly redecorated house with move-in interval ≥ 3 months	19(25.3)	5 (11.4)	
**Indoor combustion sources**			
Electric cooking stoves	12 (16.0)	7(15.9)	0.967
Charcoal or wood burning	0	0	
Household piped gas burning	63 (84.0)	37 (84.1)	
**Indoor floor decoration**			
Wood-base flooring	35 (46.7)	16 (36.4)	0.389
Marble and tile paving	9 (12.0)	10 (22.7)	
Carpet	2 (2.7)	2 (4.5)	
Mix of various materials	29 (38.7)	16 (36.4)	
**Indoor wall decoration**			
Wallpaper	4 (5.3)	2 (4.5)	0.934
Paint	35 (46.7)	22 (50.0)	
Mix of various materials	36 (48.0)	20 (45.5)	
**Indoor furniture and ornaments**			
Wood-base furniture	37 (49.3)	23 (52.3)	0.452
Wood and leather furniture	13 (17.3)	4 (9.1)	
Mix of various materials	25 (33.3)	17 (38.6)	
**Second-hand smoke**			
Not exposed	47 (62.7)	21 (47.7)	0.112
Exposed	28 (37.3)	23 (52.3)	
**Exposure to environmental pollutants nearby the residence**			
Cornfields and orchards	2	4	
Chemical plant	1	0	
Heavy-traffic road	25	18	
Incineration plant	0	1	
More than two facilities concomitantly	0	4	
***Special facilities combined***			
No	47 (62.7)	17 (38.6)	0.004
Heavy traffic road	25 (33.3)	18 (40.9)	
Other special facilities	3 (4.0)	9 (20.5)	
**Smoke ventilators usage when cooking**			
No	14 (18.7)	18 (40.9)	0.008
Yes	61 (81.3)	26 (59.1)	
**Exposure to household chemical products**			
Pesticides	7	2	
Disinfectants and sanitizers	1	1	
Paints, dyes and glues	1	0	
Household cleaning agents	5	2	
***Special chemicals combined***			
None	52 (69.3)	32 (72.7)	0.917
One	14 (18.7)	7 (15.9)	
Multiple	9 (12.0)	5 (11.4)	

^**a**^For abnormally distributed continuous variables, the median (interquartile) was used to describe the distribution, while the *P* values were calculated by the Mann–Whitney U-test

### Indoor air quality

There were no significant differences between the two groups in PM_2.5_ and PM_10_ concentrations. The median (interquartile) TVOC [0.400 (1.1525) vs. 0.005 (0.300) mg/m^3^, *P* < 0.001] level in cases was significantly higher than controls (Table 2). Formaldehyde in the two groups exceeded the Chinese indoor exposure limit in seven cases (15.9%) and 10 controls (13.3%), despite the lack of a significant difference between the groups. The concentrations of BTX were detected at very low levels in both groups with a median (interquartile) close to zero, but more cases than controls exceeded the limit. We did not find any difference in temperature or humidity between the two groups, and most of the subjects were recruited in spring or summer.

**Table 2:** Descriptive statistics of indoor air pollutants in the case and control groups^a^

Indoor air pollution	Reference limit value #	Case				Control				*P* value*
		Mean ± SD	Median (interquartile)	Range (min–max)	Number over reference limit (N/%)	Mean ± SD	Median (interquartile)	Range (min–max)	Number over reference limit (%)	
PM_2.5_, µg/m^3^	75 ^b^	12.364 ± 7.686	10.000 (8.750)	2.000–32.000	0	12.667 ± 14.589	8.000 (11.000)	1.000–73.000	0 (0.0)	0.085
PM_10_, µg/m^3^	150 ^c^	14.682 ± 9.383	12.000 (12.750)	2.000–38.000	0	14.787 ± 17.394	8.000 (14.000)	1.000–84.000	0	0.055
Benzene, mg/m^3^	0.11 ^c^	0.057 ± 0.190	0.005 (0.060)	0.005–1.000	4 (9.1)	0.035 ± 0.146	0.005 (0.000)	0.005–1.000	4 (5.3)	0.007
Toluene, mg/m^3^	0.2 ^c^	0.093 ± 0.185	0.005 (0.020)	0.005–1.000	14(31.8)	0.035 ± 0.110	0.005 (0.000)	0.005–0.600	8 (10.7)	0.005
Xylene, mg/m^3^	0.2 ^c^	0.088 ± 0.228	0.005 (0.016)	0.005–1.400	9 (20.5)	0.041 ± 0.130	0.005 (0.000)	0.005–0.600	7 (9.3)	0.016
Formaldehyde, mg/m^3^	0.1 ^c^	0.063 ± 0.127	0.010 (0.024)	0.005–0.609	7 (15.9)	0.074 ± 0.166	0.020 (0.035)	0.005–0.790	10 (13.3)	0.168
TVOC, mg/m^3^	0.6 ^c^	1.131 ± 1.602	0.400 (1.152)	0.005–7.027	16 (36.4)	0.218 ± 0.384	0.005 (0.300)	0.005–1.850	8 (10.7)	< 0.001
Temperature, ℃	/	24.704 ± 5.014	26.000 (6.000)	13.000–34.000	/	26.760 ± 3.813	28.000 (4.000)	15.000–34.000	/	0.09
Relative humidity, %	/	43.272 ± 9.879	40.500 (12.500)	27.000–69.000	/	46.613 ± 7.807	47.000 (10.000)	30.000–63.000	/	0.15

^a^BTX, formaldehyde and TVOC concentrations below the method of detection limit (MDL) of 0.01 mg/m^3^ were replaced with 0.005 mg/m^3^ in all analyses

^b^Indoor PM_2.5_ exposure limit was referenced from Chinese national ambient air quality standard GB3095-2012

^c^Indoor exposure limits were referenced from Chinese national GB/T 18,883–2002

^*^P value was calculated by Mann–Whitney U-test for nonnormally distributed continuous variables

Table S2 (Supplementary Information) shows the correlation analysis for these indoor air pollutants. The correlation coefficient between formaldehyde and other pollutants ranged from 0.19–0.46 with statistical significance. BTX concentrations were closely related to each other, with moderate correlations ranging from 0.55–0.70 (*P* < 0.001), which suggests that these three pollutants have similar or common sources. PM_2.5_ and PM_10_ concentrations were highly correlated, with a coefficient of 0.97 (*P* < 0.001), which indicates their common source of pollution. TVOCs, as the total concentrations of formaldehyde, BTX and other VOCs, showed no obvious correlations with PM.

### Associations between indoor air pollutant exposure levels and CHD

Associations between indoor air pollutant levels and CHD were investigated using univariate and multivariable single-pollutant regression models. Multivariable models were adjusted for variables that were significantly different in bivariate analysis, including parental age and education level, house renovation and ventilation, exposure to environmental pollutants near the residence, and smoke ventilator use when cooking. Because the BTX concentrations of most subjects in both groups were below the MDLs or not detectable, we did not estimate the ORs of BTX exposure levels in regression models due to the limited statistical power.

As shown in Table 3, a high level of TVOCs was associated with CHD in univariable analysis (COR 6.57, 95% CI 2.57–16.77) and was enhanced with an elevated AOR of 7.09 (2.10–23.88). Compared to infants with a low level of PM_10_, infants of mothers in the middle and high levels had higher odds for CHD, with CORs of 4.71 (95% CI: 1.43–15.60) and 4.70 (95% CI: 1.38–15.99), respectively, despite the wide confidence intervals. Similar to its strong correlation with PM_10_, PM_2.5_ exposure was similarly associated with CHD, with a middle-level COR of 3.78 (95% CI: 1.31–10.89) and a high-level COR of 3.32 (95% CI: 1.13–9.84) vs. the low level. We did not find any association between formaldehyde exposure level and CHD risk.

**Table 3:** Associations between indoor air pollutant exposure levels and CHD

Indoor air pollutant exposure levels	Controls N (%)	Cases N (%)	COR	AOR^a^
**TVOC**^**b**^				
Low (≤ 0.005 mg/m^3^)	38 (50.7)	9 (20.5)	Reference	Reference
Middle (0.006–0.300 mg/m^3^)	19 (25.3)	6 (15.9)	1.56 (0.50–4.82)	1.62 (0.40–6.56)
High (> 0.301 mg/m^3^)	18 (24.0)	19 (63.6)	6.57** (2.57–16.77)	7.09* (2.10–23.88)
**Formaldehyde**				
Low (≤ 0.01 mg/m^3^)	26 (34.7)	23 (52.3)	Reference	Reference
Middle (0.011–0.030 mg/m^3^)	25 (33.3)	9 (20.5)	0.41 (0.16–1.05)	0.73 (0.22–2.43)
High (> 0.031 mg/m^3^)	24 (32.0)	12 (27.3)	0.56 (0.23–1.38)	0.82 (0.25–2.68)
**PM**_**2.5**_				
Low (≤ 4 µg/m^3^)	27 (36.0)	6 (13.6)	Reference	Reference
Middle (5–13 µg/m^3^)	25 (33.3)	21 (47.7)	3.78* (1.31–10.89)	2.89 (0.77–10.88)
High (> 13 µg/m^3^)	23 (30.7)	17 (38.6)	3.32* (1.13–9.84)	3.26 (0.84–12.71)
**PM**_**10**_				
Low (≤ 5 µg/m^3^)	24 (32.0)	4 (9.1)	Reference	Reference
Middle (6–14 µg/m^3^)	28 (37.3)	22 (50.0)	4.71* (1.43–15.60)	5.00 (1.12–22.31)
High (> 14 µg/m^3^)	23 (30.7)	18 (40.9)	4.70* (1.38–15.99)	5.36 (1.13–25.44)

^a^Adjusted for maternal and paternal age, maternal and paternal education level, house renovation and ventilation (category), exposure to environmental pollutants near the residence (category), and smoke ventilator usage when cooking

^b^As the TVOC concentration was below the MDL in more than one-third of the control population, TVOC exposure levels were divided into low (equal or lesser than the MDL value), middle (below the median detected value of the control distribution), and high (above the median detected value of the control distribution) groups

^*^Adjust *p* value (Benjamin-Hochberg correction) < 0.05, **Adjust *p* value (Benjamin-Hochberg correction) < 0.01 vs. control

Due to the limited number of participants in the CHD subgroups, further analysis was performed to compare the cases and controls when these pollutant exposure levels were dichotomized into two levels as described in the methods section. The association with high TVOC levels was also present in the subgroups of septal defects in the context of the recategorised low level [AOR 18.62 (95% CI: 3.01–115.20)] but not in the subgroup of right-sided obstructions and conotruncal defects (Table S3). Other pollutants, including formaldehyde, PM_2.5_ and PM_10_, did not show any statistically significant association with CHD subtypes in the context of this recategorised binary exposure level.

### Effect of the association of CHD with indoor air pollutant exposure levels in diverse residential environments

We chose several residential-level environmental factors that correlated with indoor air pollutant exposure based on the previous literature. Table 4 shows the effects of air pollutants on CHD in the offspring of all participants and stratified by house renovation, smoke ventilator usage, and exposure to environmental pollutants near the residence. Due to the limited sample size, recategorised binary exposure levels of pollutants were used to compare the ORs for CHD in different stratifications.

**Table 4:** Effect of the association of CHD with indoor air pollutant exposure levels by stratified status of house renovation, smoke ventilator usage and exposure to environmental pollutants near the residence

	Living in a newly renovated house				Use of smoke ventilators when cooking				Exposure to environmental pollutants near the residence			
	No (*n* = 84)		Yes (*N* = 35)		No (*n* = 32)		Yes (*N* = 87)		None (*n* = 64)		Heavy-traffic road (*N* = 43)	
	N, controls/cases	AOR^a^	N, Controls/cases	AOR^a^	N, controls/cases	AOR^b^	N, controls/cases	AOR^b^	N, controls/cases	AOR^c^	N, controls/cases	AOR^c^
**TVOC**												
Low (≤ 0.30 mg/m^3^)	42/12	Reference	15/4	Reference	12/7	Reference	45/9	Reference	39/5	Reference	16/8	Reference
High (> 0.30 mg/m^3^)	12/18	5.43 (1.44–20.55)	6/10	9.23 (1.17–73.00)	2/11	13.10 (0.73–234.81)	16/17	5.19*(1.50–17.93)	8/12	31.37* (3.21–306.6)	9/10	2.65 (0.57–12.35)
**Formaldehyde**												
Low (≤ 0.03 mg/m^3^)	37/21	Reference	14/11	Reference	9/11	Reference	42/21	Reference	30/13	Reference	18/13	Reference
High (> 0.03 mg/m^3^)	17/9	1.68 (0.45–6.23)	7/3	0.50 (0.06–4.10)	5/7	0.68 (0.06–8.31)	19/5	0.72 (0.18–2.84)	17/4	0.40 (0.06–2.78)	7/5	0.76 (0.12–5.03)
**PM**_**2.5**_												
Low (≤ 13 µg/m^3^)	40/16	Reference	12/11	Reference	6/13	Reference	46/14	Reference	29/13	Reference	20/8	Reference
High (> 13 µg/m^3^)	14/14	2.32 (0.70–7.75)	9/3	0.53 (0.09–3.33)	8/5	0.16 (0.01–1.83)	15/12	4.27 (1.22–14.94)	18/4	0.01 (0–1.16)	5/10	6.97(1.26–38.38)
**PM**_**10**_												
Low (≤ 14 µg/m^3^)	40/16	Reference	12/10	Reference	6/12	Reference	46/14	Reference	29/13	Reference	20/8	Reference
High (> 14 µg/m^3^)	14/14	2.32 (0.70–7.75)	9/4	0.53 (0.09–3.33)	8/6	0.16 (0.01–1.83)	15/12	4.27 (1.22–14.94)	18/4	0.01 (0–1.16)	5/10	6.97(1.26–38.38)

^a^Adjusted for maternal and paternal age, maternal and paternal education level, exposure to environmental pollutants near the residence (category), and smoke ventilator usage when cooking

^b^Adjusted for maternal and paternal age, maternal and paternal education level, house renovation and ventilation (category), and exposure to environmental pollutants near the residence (category)

^c^Adjusted for maternal and paternal age, maternal and paternal education level, house renovation and ventilation (category), and smoke ventilator usage when cooking

The low-level group served as the reference

^*^Adjust *p* value (Benjamin-Hochberg correction) < 0.05 vs. control

Among participants living in newly redecorated houses, high TVOC exposure was associated with the risk of CHD (AOR 9.23, 95% CI: 1.17–73.00) despite without significant statistical difference. The AOR of high TVOC for CHD was estimated as 5.19 (95% CI: 1.50–17.93) in houses that used smoke ventilators when cooking, and the AOR value tended to be higher in houses without the use of smoke ventilators, but the difference was not statistically significant (AOR 13.1, 95% CI: 0.73–234.81, *p* = 0.08). Associations of high PM_2.5_ and PM_10_ exposure levels with the risk of CHD were not found in participants in diverse residential environments in our study. However, the CHD risk associated with most air pollutants was not statistically significant in participants with no house renovation, no use of smoke ventilators or no exposure to environmental pollutants near the residence.

### Interaction effects of exposure to TVOCs and PM_2.5_ on the risks of CHD

We analysed interactions between TVOC and PM_2.5_ using pairwise combinations modifying the risk for CHD (Table 5). We observed a joint association AOR of 8.99 (95% CI: 2.05–39.41) for high TVOCs with high PM_2.5_ compared to low TVOCs and low PM_2.5_. There was a statistically significant multiplicative interaction between TVOCs and PM_2.5_ (AOR 6.99, 95% CI: 1.75–27.85), which suggests that high TVOCs and high PM_2.5_ interact synergistically to increase the risk of CHD. Although an RERI value of 5.49 was calculated in the additive model, which indicates a likely positive interaction of TVOCs and PM_2.5_, this evidence was weak, as evidenced by the wide confidence interval crossing 0 (95% CI: -8.99–19.97).

**Table 5:** Interaction effects of exposure to TVOCs and PM_2.5_ on the risks of CHD

Exposure	N, controls/cases	AOR^a^ (95% CI)
Low TVOC and low PM_2.5_	40/12	Reference
High TVOC and low PM_2.5_	12/15	3.70 (0.93–14.65)
Low TVOC and high PM_2.5_	17/4	0.80 (0.18–3.66)
High TVOC and high PM_2.5_	6/13	8.99* (2.05–39.41)
Cross-product for interaction		6.99* (1.75–27.85)
RERI on the additive scale		5.49 (-8.99–19.97)
AP on the additive scale		0.61 (-0.25–1.47)

Low TVOC was defined as a TVOC concentration ≤ 0.30 mg/m^3^, while high TVOC was defined as a TVOC concentration > 0.30 mg/m^3^; low PM_2.5_ was defined as a PM_2.5_ concentration ≤ 13 µg/m^3^, while high PM_2.5_ was defined as a PM_2.5_ concentration > 13 µg/m^3^

^a^Adjusted for maternal and paternal age, maternal and paternal education level, house renovation and ventilation (category), exposure to environmental pollutants near the residence (category), and smoke ventilator usage when cooking. The low-level group served as the referenc

^*^Adjust *p* value (Benjamin-Hochberg correction) < 0.05 vs. control

## Discussion

Reports linking environmental exposures to birth defects have steadily increased [11, 19, 21]. However, the inability to routinely identify indoor environmental exposures during pregnancy, the difficulty in quantifying these exposures and maternal recall biases limit the ability to determine causal relationships. Indoor air pollution, compared to atmospheric pollution, is characterized by its complexity, ubiquity, and persistence, and it results in progressive and cumulative effects on health risk, especially in susceptible pregnant women [28]. This research suggested that most pregnant women spend more time indoors. Therefore, indoor air quality data are more important for analysing these effects.

The current study determined the associations between CHD in offspring and maternal exposure to indoor air pollution during pregnancy as measured in field-based investigations with a case–control design. We observed that exposure to indoor TVOCs, PM_2.5_ and PM_10_ at high levels during pregnancy resulted in increased risks for CHD and the occurrence of some major subtype in offspring. Some household activities, including house renovation and smoke ventilator use when cooking may affect the risks of CHD associated with indoor air pollutants. Our results also suggested that maternal exposure to high levels of TVOCs and PM_2.5_ interacted synergistically to increase the risk of CHD. These results support the hypothesis that maternal exposure to indoor air pollution has adverse effects on foetal cardiac development.

VOCs and PM are major indoor air pollutants that were widely studied for their adverse effects on cardiovascular [12] and pulmonary health [13] in the general population, but limited data are available in pregnant women and their foetuses. Some volatile organic solvents (e.g., trichloroethylene (TCE)) quickly evaporate and are preserved in the atmosphere, soil and ground water, and these solvents are sources of indoor pollutant exposure via inhalation, ingestion and skin contact [29]. One study indicated that TCE was likely a risk factor for CHD and reported a threefold increased CHD risk in mothers presumably exposed to TCE compared to the risk in nonexposed mothers [30]. Chang et al. [31] indicated that elevated exposure to TVOCs during the prenatal period may adversely influence early postnatal growth. A previous study [19] assessed the effects of ambient air pollutants on CHD and found that effect estimates of cardiac atrial septal defects for the first trimester were significantly increased with continuous and categorical PM_10_ exposure high exposures were compared to low exposures. However, exposure assessed by air pollution monitoring stations as a proxy for personal exposure resulted in smaller effect estimates at the municipal level than the use of individual assessments of exposure [32]. To our knowledge, no prior study evaluated the effects of indoor air pollution on foetal CHD using individual maternal exposure data, and most studies were limited by self-reported or occupational exposure assessments.

Indoor air pollutant levels measured in our study participants were generally lower than the levels reported in previous studies. The mean value of formaldehyde was 0.063 mg/m^3^ for cases and 0.074 mg/m^3^ for controls in our study, which are lower than the reported mean value of 0.175 mg/m^3^ in general houses in Harbin, China [33] and 81.6 μg/m^3^ in houses for pregnant women in Korea [31]. BTX concentrations in most houses were below the MDLs or were not detected. For total VOCs, a reported household mean value of 0.411 mg/m^3^ (range 0.28–0.48 mg/m^3^) [33] was similar to our median concentration in cases (0.400 mg/m^3^) but far exceeded the concentration of controls (0.005 mg/m^3^). The median TVOC concentration in cases was also higher than the prenatal exposure value of 284.2 μg/m^3^ in Chang’s research [31]. One study in Taipei, China recorded household indoor PM_10_ and PM_2.5_ mean concentrations of 41 µg/m^3^ (ranging from 7.8–99.4 µg/m^3^) and 25.5 µg/m^3^ (range 9.5–80.5 µg/m^3^), respectively, which were higher than the values in our control participants (Table 2). According to the Chinese National Air Quality Standards GB/T 18,883 and GB3095, the rates of exceeding the current reference value in both groups were below 20% for most indoor air pollutants except for TVOCs and toluene in the case group (36.4% and 31.8%, respectively).

Although the levels of maternal exposure to indoor air pollutants in our study were generally lower than previous studies of the general population and below the Chinese national standard reference values, our results indicated that the risk of CHD occurrence in offspring may be associated with maternal high TVOC, PM_2.5_ and PM_10_ exposure levels, even at low concentrations. Compared to mothers with low TVOC levels (≤ 0.30 mg/m^3^), mothers exposed to high TVOC levels (> 0.30 mg/m^3^) had a 5.92 times higher risk for total CHD (AOR 5.92, 95% CI: 2.01–17.40), a 18.62 times higher risk for septal defects (AOR 18.62, 95% CI: 3.01–115.20) after adjusting for confounders (Table S3). We found that PM_2.5_ concentrations above 5 µg/m^3^ and PM_10_ concentrations above 6 µg/m^3^ were associated with higher odds for total CHD than their respective low concentrations levels (Table 3). However, the target VOCs, such as formaldehyde and BTX, were not associated with CHD risk in our study, likely because newly presented or complicated organic compounds that contributed to the TVOC concentration but were not detected in our research, played a role in CHD occurrence. Despite the imprecise effect estimates and undefined dose–effect relationships resulting from the small sample size, it is concerning that the current reference values in Chinese indoor air quality standards may lead to an underestimation of the risks for CHD associated with pollutants in pregnant women.

It is difficult to offer a plausible biological mechanism for the occurrence of developmental toxicity and cardiac teratogenicity as a result of air pollutant exposure. Potential mechanisms underlying air pollutant-induced teratogenicity were reported, including chromosome and DNA damage (genotoxicity), oxidative stress, altered levels and/or functions of enzymes, hormones and proteins, apoptosis, and toxicogenomic and epigenomic effects (such as DNA methylation) [34, 35]. Some studies suggested [36] that maternal exposure to air pollution influenced endothelial function and blood viscosity, which would alter maternal–placental oxygen and nutrient exchanges and affect foetal development. The association that we observed may be attributable to a joint effect of several air pollutants based on various biological mechanisms.

Concentrations of indoor air pollutants exhibit individual variation due to complex and diverse indoor or outdoor sources, room temperature and humidity, living habits or the use of appliances that reduce or increase the pollutant concentration [37].

TVOCs consist of various VOCs, such as formaldehyde, trichloroethylene, benzene series, and hydrocarbon compounds, and are primarily attributed to indoor sources, including building materials, household chemical products, and combustion processes causing smoke [38]. High concentrations of VOCs, which may be emitted from various materials, such as paints, dyes, adhesives, solvents, boards and plywood, are more often reported in new residential buildings or renovated dwellings [37, 39, 40]. A case–control study in China described that maternal exposure to house renovations increased the risk of CHD, and this relationship was stronger for women who had moved into a newly decorated house [21]. Although there was insufficient evidence of the effects of traditional decorative materials, such as floors, wall decorations and furniture, on CHD risk in our results, we observed a likely higher TVOC exposure odds for CHD in newly renovated houses (AOR 9.23, 95% CI: 1.17–73.00) than houses without renovation (AOR 5.43, 95% CI: 1.44–20.55) (Table 4). This finding suggests that people are prone to using traditional decorative materials that are environmentally friendly but that more new-style or complicated ornaments in newly renovated houses that were not reported in our study may contribute to indoor TVOC pollution. For subjects in newly renovated houses, fewer CHD occurred in subjects living in houses with more than a 3-month moving-in interval, which suggests that renovation-related volatile air pollutants are time dependent and decrease over time.

Outdoor sources resulting from the oil and gas industry, transport emissions and biogenic emissions affect indoor TVOC concentrations via air exchange [41]. Human activities are significant sources of indoor air pollution. Natural and mechanical ventilation improve the levels of chemical pollutants in indoor air [37, 42]. Second-hand smoke was not associated with CHD in our study, but smoke ventilator use during cooking tended to modify the effect of TVOCs on CHD, which suggests that the improvement in indoor air quality had a positive impact on lowering CHD risks. Although some studies indicated that the levels of indoor PM_2.5_ resulted from pan frying in kitchens [43], our study did not show that smoke ventilator use in cooking modified the effect of PM on CHD. Traffic-related emissions from the outdoors did not interfere with the effect of indoor TVOCs and PMs on CHD.

However, the present study could not address the contribution of indoor air pollutants from different sources directly due to their complexities. People's living habits and residential environments also vary individually and affect the level of exposure to indoor air pollutants. More detailed investigations and comprehensive sampling detections are needed in further studies.

In contrast to one study that revealed a negative correlation between indoor TVOC and PM concentrations (PM_0.5_ and PM_1_) [37], TVOC concentrations showed no obvious correlations with PM_2.5_ or PM_10_ in our study, which suggests that they originated from different sources. However, we found that PM exposure had different associations with TVOCs and CHD. Compared to co-exposure to low levels of TVOCs (≤ 0.30 mg/m^3^) and PM_2.5_ (≤ 13 µg/m^3^), co-exposure to high levels of TVOCs (> 0.30 mg/m^3^) and PM_2.5_ (> 13 µg/m^3^) was associated with an 8.99 times higher risk for CHD. These results suggest that co-exposure to TVOCs and PM_2.5_ had a certain synergistic effect on CHD occurrence (Table 5). An animal experiment suggested that exposure to a combination of PM_2.5_ and formaldehyde resulted in increased lung damage in mice with allergic asthma as a result of oxidative stress, immunogenic responses and neurogenic responses [44]. The biological mechanisms for the joint effect of indoor air pollutants on foetal cardiac development must be further investigated.

Despite constant public concern about indoor air quality, China's national standards, GB/T 18,883–2002 and GB3095-2012, were successively enacted and set guideline values for formaldehyde, BTXs, TVOCs, PM_10_ and PM_2.5_. However, a specific threshold for susceptible populations, especially pregnant women, was not defined in China, which is likely due to a lack of information on exposure and risk assessment. Our research initially indicated that indoor air pollution was associated with foetal CHD, but the exact cut-off value for CHD occurrence must be confirmed in an abundant sample using a sophisticated design. To the best of our knowledge, the current study is the first study to examine the CHD-related effects of personal maternal air pollution exposure, rather than data obtained from air quality monitoring stations in many previous studies [8, 11, 19]. Personal measurements are generally considered a more accurate representation of exposure levels.

The main limitation of our study was the small sample size, which resulted in limited statistical power. The CIs for less common exposures were wide. The problem of insufficient sample size was especially prominent for some subtypes, such as anomalous pulmonary venous return. The real statistical power may be affected by the small samples for the specific subtypes. Therefore, we primarily demonstrated associations between maternal exposure to indoor air pollution and total CHD in our study population. Given the wide intervals, we could not provide sufficiently reliable results for hazard effects of indoor air pollutants exposure on CHD. Second, air samples were collected from participants at a median of 25 gestational weeks after the critical period for cardiac development, which occurs during the first three months of gestation [14], and this timing may have introduced nondifferential misclassification and decreased the accuracy of the exposure assessment. Although the volatility of indoor air pollution may have resulted in low detection values at sampling time and misclassify exposure level for some participants, the collection of data and air samples in this study were based on unaltered lifestyles and immobile living environments, and the results may reflect the relatively quantifiable exposure within the pregnancy period for the case–control design. Our survey timing was much earlier than most previous reports, which generally collected personal exposure air samples during the third trimester of pregnancy [31, 45] or after delivery [46]. Third, more measured indoor and outdoor pollutant concentrations are needed to calculate indoor/outdoor ratios and confirm the source of pollutants. Although we collected prenatal information on sociodemographics, reproductive history and periconceptional health status based on previous literature [3–7] and adjusted the analysis for several covariates, we cannot exclude potential confounding by unmeasured or unknown factors. In addition, we adopted a combination of questionnaire survey and field investigation to minimize the effects of recall bias as much as possible. Dwelling-related self-report questionnaires were verified in a field survey. The concentrations of formaldehyde, BTX, TVOCs, and PMs in households were objectively monitored in field investigations. More detailed investigations and comprehensive sampling detections are needed in further studies. Due to the small sample size, the number of determinants included in the models was restricted. Therefore, we did not assess the risks in the multipollutant model.

## Conclusions

Because increasing evidence suggests that ambient air pollution, which has teratogenic properties, results in a serious threat to humans at birth and may increase the risk of CHD, indoor air quality has become a public health concern in developed eastern China. Our findings indicated adverse effects of TVOCs, PM_2.5_ and PM_10_ during pregnancy on the outcome of foetal CHD, even at low exposure levels. These effects may have been enhanced for pregnant women living in a newly renovated house but were mitigated by household use of smoke ventilators when cooking. Our results also suggested a synergistic interaction of maternal exposure to TVOCs and PM_2.5_ in the risk for CHD. Our study results provide useful evidence for the development of interventions to improve indoor air quality for pregnant women.

## Supplementary Information

**Additional file 1: Table S1.** Basic characteristics (sociodemographics, reproductive history, and periconceptional health status) of the study subjects. **Table S2.** Pearson correlation coefficients for indoor air pollution. **Table S3.** Effect of indoor air pollutant exposure levels on CHD subtypes.^a^.

## Data Availability

The datasets generated and/or analysed during the current study are not publicly available due to data privacy issues but are available from the corresponding authors (KS and YW) on reasonable request.

## Abbreviations

CHD: Congenital heart disease
PM: Particulate matter
PM_10_: Particulate matter less than 10 microns in diameter
PM_2.5_: Particulate matter less than 2.5 microns in diameter
VOCs: Volatile organic compounds
BTX: Benzene, toluene, xylene
TVOCs: Total volatile organic compounds
GC-FID: Gas chromatography with flame ionization detection
MBTH: 3-Methyl-2-benzonthiazolinone hydrazine
MDLs: Method detection limits
COR: Crude odds ratio
CI: Confidence interval
AOR: Adjusted odds ratio
RERI: Relative excess risk due to interaction
AP: Attributable proportion
