# Supplementary material for: Association between maternal exposure to indoor air pollution and offspring congenital heart disease: a case–control study in East China
Source: BMC Public Health. 2022 Apr 15;22:767. doi: 10.1186/s12889-022-13174-0 (PMC9013107; doi:10.1186/s12889-022-13174-0)
Supplement: Supplementary file 1 — Additional file 1: Table S1. Basic characteristics (sociodemographics, reproductive history, and periconceptional health status) of the study subjects. Table S2. Pearson correlation coefficients for indoor air pollution. Table S3. Effect of indoor air pollutant exposure levels on CHD subtypes.a. [file 12889_2022_13174_MOESM1_ESM.docx]

**Table S1. Basic characteristics (sociodemographics, reproductive history, and periconceptional health status) of the study subjects**

| **Characteristics** | **Controls**  **(N=75)** | **Cases**  **(N=44)** | **p Value*** |
| --- | --- | --- | --- |
|  | n (%) | n (%) |  |
| **Maternal age (years)** |  |  |  |
| <25 | 1 (1.3) | 4 (9.1) | 0.105 |
| 25–29 | 20 (26.7) | 16 (36.4) |  |
| 30-34 | 40 (53.3) | 18 (40.9) |  |
| 35+ | 14 (18.7) | 6 (13.6) |  |
| **Paternal age (years)** |  |  |  |
| <25 | 0 (0) | 1 (2.3) | 0.002 |
| 25–29 | 12 (16.0) | 20 (45.5) |  |
| 30-34 | 38 (50.7) | 13 (29.5) |  |
| 35+ | 25 (33.3) | 10 (22.7) |  |
| **Maternal education level** |  |  |  |
| Low (compulsory only) | 1 (1.3) | 6 (13.6) | 0.001 |
| Medium (completed high school) | 8 (10.7) | 11 (25.0) |  |
| High (tertiary or higher) | 66 (88.0) | 27 (61.4) |  |
| **Paternal education level** |  |  |  |
| Low (compulsory only) | 3 (4.0) | 10 (22.7) | 0.003 |
| Medium (completed high school) | 6 (8.0) | 6 (13.6) |  |
| High (tertiary or higher) | 66 (88.0) | 28 (63.6) |  |
| **Maternal occupation** |  |  |  |
| Individual operator | 9 (12.0) | 4 (9.1) | 0.382 |
| Professional and technical staff | 12 (16.0) | 3 (6.8) |  |
| Manicurist | 0 (0) | 1 (2.3) |  |
| Office staff | 28 (37.3) | 19 (43.2) |  |
| Marketing, sales and customer service staff | 15 (20.0) | 7 (15.9) |  |
| Full-time housewife | 11 (14.7) | 10 (22.7) |  |
| **Residence** |  |  |  |
| Urban | 72 (96.0) | 42 (95.5) | 0.886 |
| Rural | 3 (4.0) | 2 (4.5) |  |
| **Parity** |  |  |  |
| Multiparous | 28 (37.4) | 16 (36.4) | 0.916 |
| Primiparous | 47 (62.7) | 28 (63.6) |  |
| **Folic acid supplementation** |  |  |  |
| Never | 11 (14.7) | 5 (11.4) | 0.845 |
| Occasionally/irregularly | 18 (24.0) | 10 (22.7) |  |
| Frequently/regularly | 46 (61.3) | 29 (65.9) |  |
| **Multivitamin supplementation** |  |  |  |
| Never | 31 (41.3) | 25 (56.8) | 0.249 |
| Occasionally/irregularly | 12 (16.0) | 6 (13.6) |  |
| Frequently/regularly | 32 (42.7) | 13 (29.5) |  |
| **[Diabetes](javascript:;)** |  |  |  |
| No | 67 (89.3) | 39 (88.6) | 0.906 |
| Yes | 8 (10.7) | 5 (11.7) |  |
| **Maternal alcohol consumption** |  |  |  |
| No | 55 (73.3) | 32 (72.7) | 0.730 |
| Occasionally/irregularly | 19 (25.3) | 12 (27..3) |  |
| Frequently/regularly | 1 (1.3) | 0 |  |
| **Maternal smoking** |  |  |  |
| No | 73 (97.3) | 43 (97.7) | 0.895 |
| Occasionally | 2 (2.7) | 1 (2.3) |  |

*****P value was calculated with chi-square or Fisher exact tests.

**Table S2. Pearson correlation coefficients for indoor air pollution**

|  | PM_2.5_ | PM_10_ | Benzene | Toluene | Xylene | Formaldehyde | TVOC |
| --- | --- | --- | --- | --- | --- | --- | --- |
| PM_2.5_ | 1.00 |  |  |  |  |  |  |
| PM_10_ | 0.97** | 1.00 |  |  |  |  |  |
| Benzene | 0.13 | 0.15 | 1.00 |  |  |  |  |
| Toluene | -0.02 | -0.01 | 0.70** | 1.00 |  |  |  |
| Xylene | 0.03 | 0.03 | 0.55** | 0.55** | 1.00 |  |  |
| Formaldehyde | 0.30** | 0.27** | 0.46** | 0.19* | 0.40** | 1.00 |  |
| TVOC | 0.17 | 0.16 | 0.18* | 0.17 | 0.27** | 0.27** | 1.00 |

Correlations were calculated with the Spearman rank correlation test; * P<0.05; ** p<0.01

**Table S3. Effect of indoor air pollutant exposure levels on CHD subtypes. ^a^**

|  | TVOC | | |  | Formaldehyde | | |  | PM_2.5_ | | |  | PM_10_ | | |
| --- | --- | --- | --- | --- | --- | --- | --- | --- | --- | --- | --- | --- | --- | --- | --- |
|  | Low  (≤0.30 mg/m^3^) | High  (>0.30 mg/m^3^) | |  | Low  (≤0.03 mg/m^3^) | High  (>0.03 mg/m^3^) | |  | Low  (≤13 µg/m^3^) | High  (>13 µg/m^3^) | |  | Low  (≤14 µg/m^3^) | High  (>14 µg/m^3^) | |
|  | N | N | AOR  (95% CI) |  | N | N | AOR (95% CI) |  | N | N | AOR  (95% CI) |  | N | N | AOR  (95% CI) |
| **Controls** (n=75) | 57 | 18 |  |  | 51 | 24 |  |  | 52 | 23 |  |  | 52 | 23 |  |
| **Cases** |  |  |  |  |  |  |  |  |  |  |  |  |  |  |  |
| Total (n=44) | 16 | 28 | 5.92*  (2.01-17.40) |  | 32 | 12 | 0.94  (0.32-2.75) |  | 27 | 17 | 1.63  (0.60-4.39) |  | 26 | 18 | 1.63  (0.61-4.40) |
| Septal defects (n=21) | 7 | 14 | 18.62*  (3.01-115.20) |  | 17 | 4 | 0.42  (0.07-2.45) |  | 12 | 9 | 1.35  (0.33-5.56) |  | 10 | 10 | 1.58  (0.40-6.16) |
| Right-sided obstructions (n=15) | 5 | 10 | 15.14  (1.75-131.10) |  | 11 | 4 | 0.45  (0.07-2.82) |  | 10 | 5 | 1.16  (0.17-7.86) |  | 10 | 5 | 1.16  (0.17-7.86) |
| Conotruncal defects (n=12) | 7 | 5 | 5.06  (0.87-29.42) |  | 8 | 4 | 2.15  (0.41-11.19) |  | 6 | 6 | 3.09  (0.69-13.77) |  | 6 | 6 | 3.09  (0.69-13.77) |

a Adjusted for maternal and paternal age, maternal and paternal education level, house renovation and ventilation (category), exposure to environmental pollutants near the residence (category), and smoke ventilator usage when cooking. The low-level group served as the reference.

* Adjust p value (Benjamin-Hochberg correction) < 0.05 vs. control.
